# Analytical Validation of Telomere Analysis Technology® for the High-Throughput Analysis of Multiple Telomere-Associated Variables

**DOI:** 10.1186/s12575-019-0115-z

**Published:** 2020-01-15

**Authors:** Nuria de Pedro, María Díez, Irene García, Jorge García, Lissette Otero, Luis Fernández, Beatriz García, Rut González, Sara Rincón, Diego Pérez, Estefanía Rodríguez, Enrique Segovia, Pilar Najarro

**Affiliations:** Life Length SL, Miguel Ángel 11, 28010 Madrid, Spain

**Keywords:** Telomere length, Analytical validation, HT-Q-FISH, Laboratory developed test, epidemiology

## Abstract

**Background:**

A large number of studies have suggested a correlation between the status of telomeres and disease risk. High-throughput quantitative fluorescence in situ hybridization (HT Q-FISH) is a highly accurate telomere measurement technique that can be applied to the study of large cell populations. Here we describe the analytical performance testing and validation of Telomere Analysis Technology (TAT®), a laboratory-developed HT Q-FISH-based methodology that includes HT imaging and software workflows that provide a highly detailed view of telomere populations.

**Methods:**

TAT was developed for the analysis of telomeres in peripheral blood mononuclear cells (PBMCs). TAT was compared with Terminal Restriction Fragment (TRF) length analysis, and tested for accuracy, precision, limits of detection (LOD) and specificity, reportable range and reference range.

**Results:**

Using 6 different lymphocyte cell lines, we found a high correlation between TAT and TRF for telomere length (R^2^ ≥ 0.99). The standard variation (assay error) of TAT was 454 base pairs, and the limit of detection of 800 base pairs. A standard curve was constructed to cover human median reportable range values and defined its lower limit at 4700 bp and upper limits at 14,400 bp. Using TAT, up to 223 telomere associated variables (TAVs) can be obtained from a single sample. A pilot, population study, of telomere analysis using TAT revealed high accuracy and reliability of the methodology.

**Conclusions:**

Analytical validation of TAT shows that is a robust and reliable technique for the characterization of a detailed telomere profile in large cell populations. The combination of high-throughput imaging and software workflows allows for the collection of a large number of telomere-associated variables from each sample, which can then be used in epidemiological and clinical studies.

## Background

Telomeres are complexes of repetitive DNA and proteins at the end of chromosomes. They play major roles in the physiology of chromosome replication and maintenance and in the prevention of chromosome fusion during mitosis [1]. Telomerase is the enzyme that synthesizes telomeres, therefore countering their gradual shortening in each cell cycle. Telomerase is expressed and active in germ cells, stem cells, and some somatic cells, but most tissues lack or have very minimal telomerase activity [2, 3]. In addition to chromosome replication, oxidative stress and inflammation can cause DNA damage leading to telomere shortening [4]. It is estimated that human telomere length (TL) is reduced by 20–40 bp annually in leukocytes [5]. When telomeres reach a critical length, the cell activates DNA damage checkpoints and cellular senescence or apoptosis occurs [6].

Since the seminal work suggesting an association between telomere shortening and aging [7] or stress [8], there has been renewed interest in analyzing TL as a biomarker of cellular response to various conditions, diseases and environmental factors. The current view suggests that, more than a passive biomarker of aging, telomeres could play an active role in pathologies as diverse as cardiovascular disease and cancer [9]. Although many studies have reported an association between TL and various conditions and even mortality, in many cases the substantial intrinsic variability of the telomere measurement techniques employed made difficult to reach definite conclusions. Well defined, reproducible, and robust workflows are therefore required that can efficiently assess telomere features and be applied to examine large sample sets. These needs are especially relevant in precision medicine, which attempts to direct individualized treatment based on alterations detectable in the patient at the molecular level.

Several techniques have been developed to measure TL, and each approach has distinct advantages and disadvantages [10]. Terminal Restriction Fragment (TRF) length analysis uses Southern blotting or in-gel hybridization with a labeled probe specific for telomere DNA, providing an average TL of the total cell population. The TRF method, although considered the gold-standard in TL measurements, requires substantial amounts of DNA and is a relatively insensitive technique for detecting very short telomeres [11]. Several methodologies based on the polymerase chain reaction (PCR) have allowed the measurement of TL using small amounts of DNA. Methods based on PCR include single TL analysis to measure the specific length of a single chromosome end [12], quantitative PCR (qPCR), and monochrome multiplex quantitative PCR, which amplify telomeres to generate a ratio between the telomere and a standard single copy gene. Since these techniques can be adapted to high throughput (HT), they have often been applied to large population studies [13]. However, variability of PCR-based techniques can be substantial within and between samples (> 10%) [14] and, as with TRF only, a single value (TL average) is obtained.

Fluorescent in situ hybridization (FISH) technologies using digital microscopy (quantitative FISH, Q-FISH) or flow cytometry (flow FISH) can determine cell average TL and other parameters [15–17]. Although technically demanding and requiring fresh cell samples, these are highly accurate and can detect subtle changes in TL. HT Q-FISH is relevant because determination of individual telomeres allows for the examination of many variables, which can be looked at the same time in a large number of cells. Thus, detailed statistical calculations can be obtained on the full distribution of the telomeres measured. Of special interest is the frequency of short telomeres (< 3 kbp), as this parameter has been associated to higher mortality [18–21]. HT Q-FISH is the ideal technique for the investigation of short telomeres, and it was the technique used to verify their relative frequency as critical for cell viability and chromosome stability in mice [22, 23].

To provide a robust, standardized, and reproducible assay to support telomere studies in clinical samples and accommodate potentially large numbers of such specimens, Life Length SL has obtained accreditation through the Clinical Laboratory Improvement Amendments (CLIA) for the US, and internationally with the ISO15189 program to perform Telomere Analysis Technology (TAT). TAT is a modification of interphase HT Q-FISH which uses automated procedures on 384-well plates for large-scale studies on fixed lymphocytes. Here we report on the methods used and the results obtained during the analytical performance testing and validation of TAT. The technology was tested for accuracy, precision, limits of detection (LOD) and specificity, reportable range and range of values for reference samples. Additionally, we present a pilot study using TAT in which we used a database of > 8000 samples of a population of healthy individuals to better understand general trends of TL with gender and age.

## Results

**Fig. 1:**
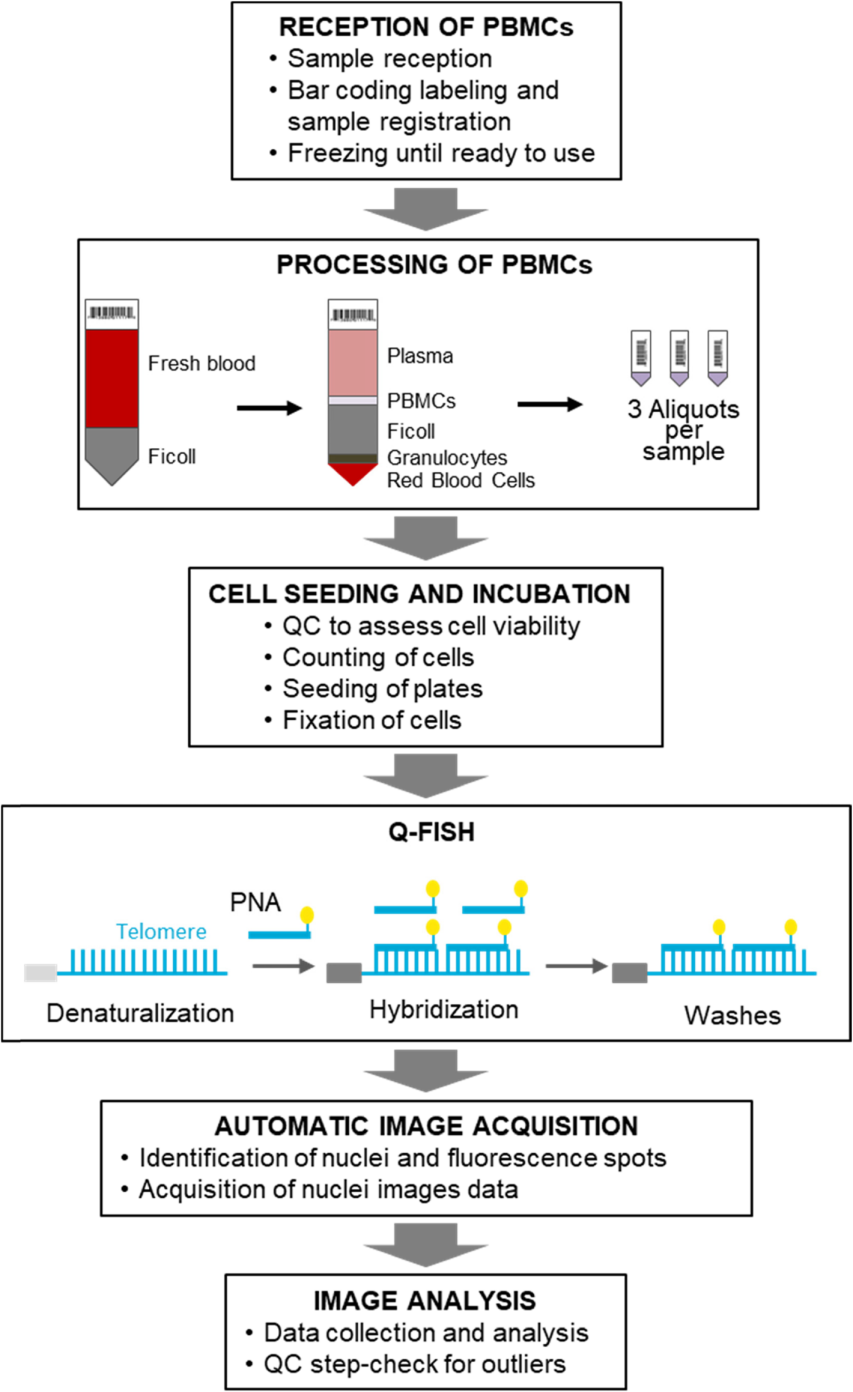
Workflows and components in the TAT system. Samples of blood requested by physicians at health centers or research laboratories are collected (10 ml) in K2 EDTA tubes and coded. The blood is shipped in < 48 h to Life Length’s laboratories for PBMC purification. After PBMC extraction, the sample is divided in three aliquots and stored in liquid-nitrogen. After thawing, cells are assessed for viability, counted, seeded on 384-well plates, and fixed. Q-FISH is carried out with a fluorescently labelled PNA probe that binds with high specificity to telomeric sequences. All steps are performed using an established documented laboratory workflow, algorithms for image analysis, and quality control

The overall laboratory workflow and components in the TAT system are depicted in Fig. 1. TAT was developed for the analysis of telomeres in peripheral blood mononuclear cells (PBMCs), which are extracted by gradient centrifugation from 10 mL samples of blood. Here, to analyze the performance of TAT, we investigated its accuracy, precision, LOD, reportable range and reference range in samples of well-described lymphocyte cell lines.

As a reference for comparison against what can be considered the “true value” we analyzed the same samples with telomere restriction fragment (TRF) analysis, the first technique developed to determine TL and the gold standard for TL determination [11, 24]. TRF allows the visualization of telomeres of all sizes in the sample, showing their asymmetrical size distribution which can then be used for quantitative determinations [11].

### Accuracy Assessment

**Fig. 2:**
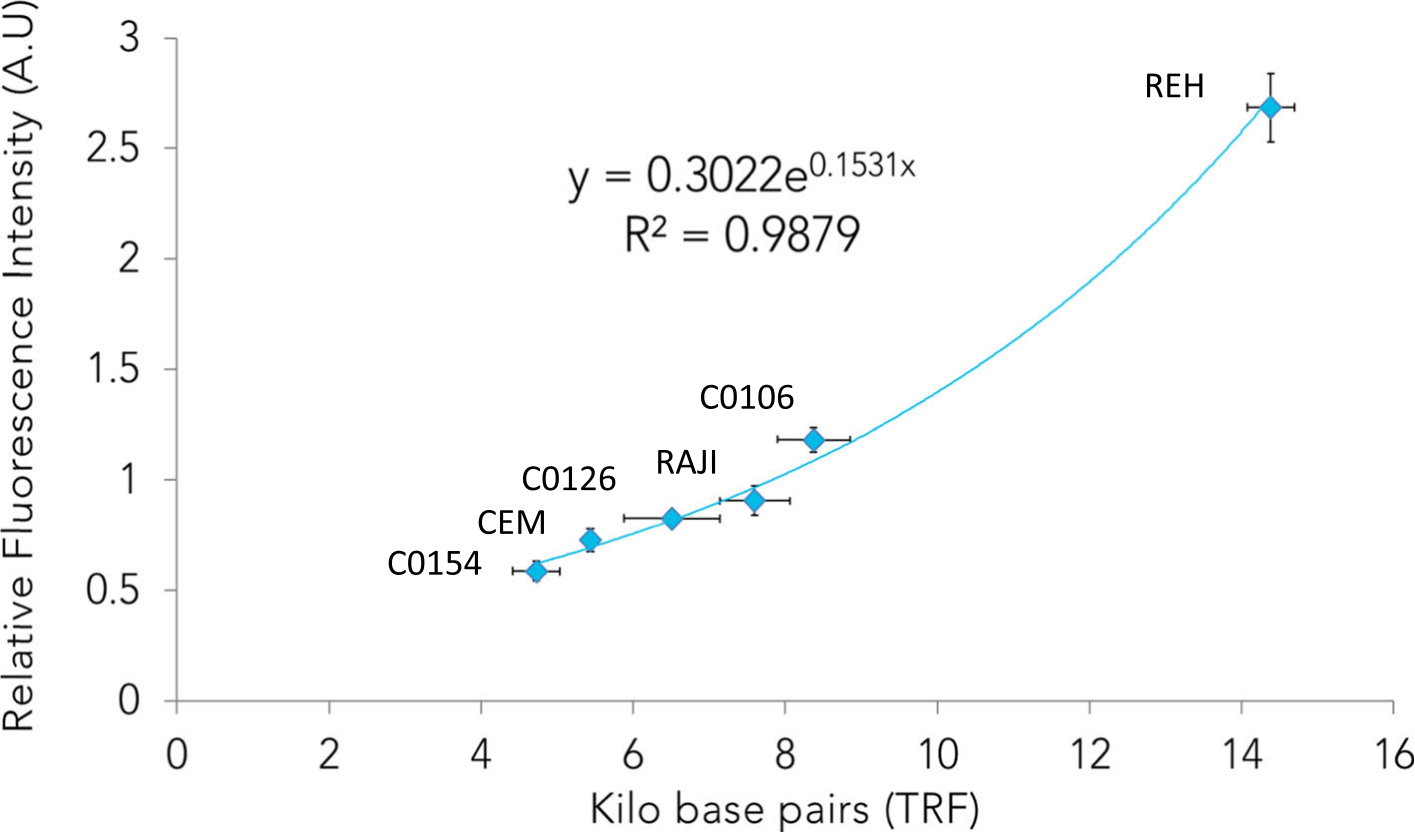
Comparison of the TL results obtained for the six control cell lines by TAT and by TRF in three independent assays. Standard errors are represented with vertical bars (for TAT) and horizontal bars (for TRF)

To evaluate the accuracy of TAT we compared the TL obtained by the TAT method with values obtained with TRF analysis. Six human lymphocyte cell lines were analyzed 3 times by TRF and by TAT to determine the correlation between TRF results (expressed in kilo base pairs or Kbp) and TAT relative fluorescence intensity values. The results obtained (Fig. 2) showed a strong correlation between these techniques (R^2^ ≥ 0.99) for the cell lines of reference. TAT results showed smaller standard variation (SD) than TRF results.

**Fig. 3:**
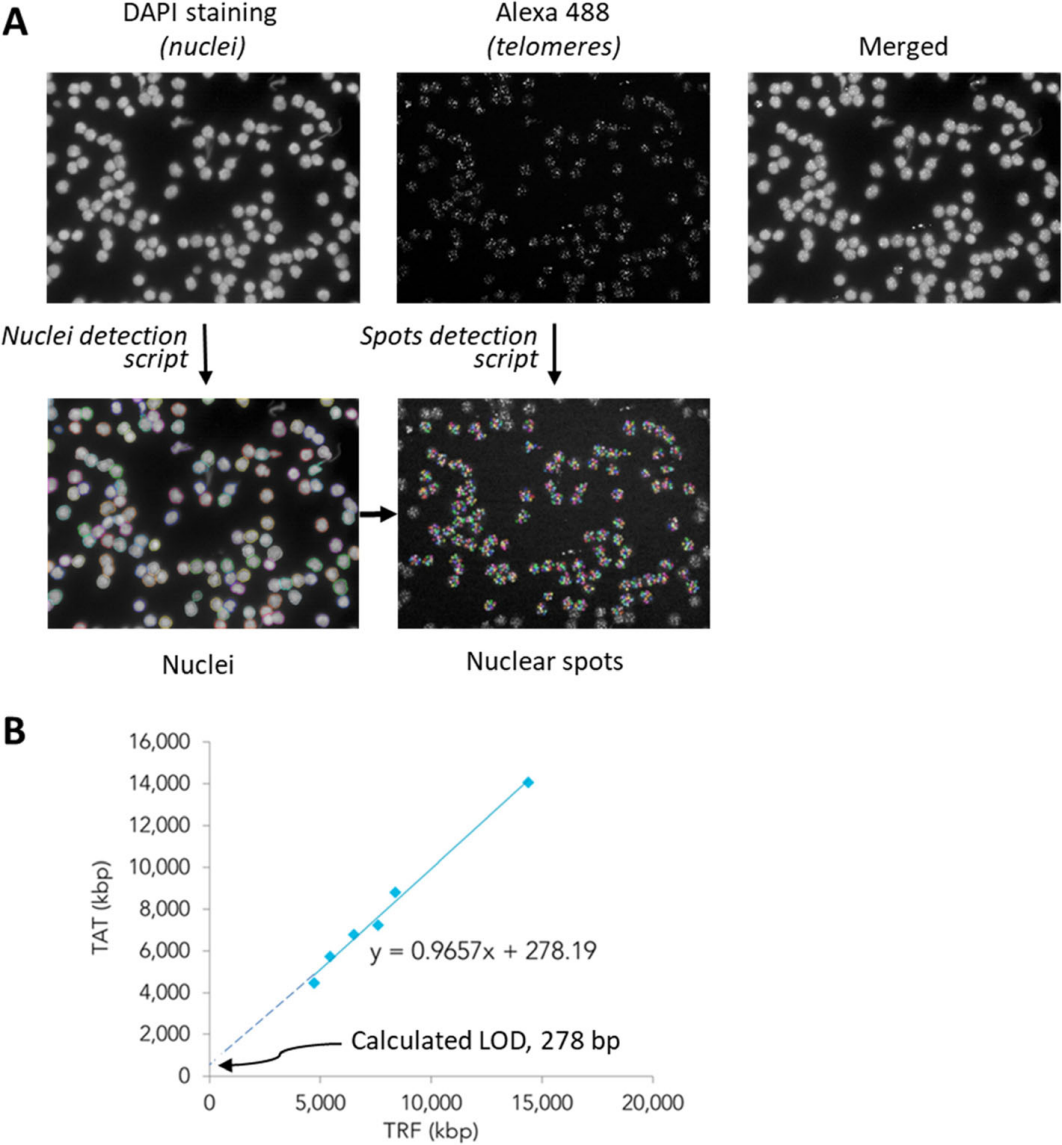
Nuclear spots detection and LOD determination. **a**. Scheme of image capture and processing by the Opera® High Content Screening System and Acapella® software. **b**. Correspondence of absolute telomere length values and LOD determination by extrapolation from the accuracy curve TAT vs TRF (Kpb to Kpb), expressed in bp that corresponds to the intersection of the linear regression curve with the y-axis

The data of the two sets of results were plotted to establish the regression equation (Fig. 3b). TAT systemic error is defined as the combination of proportional (indicated by slope) and constant (indicated by intercept) errors as shown in this regression curve. The results show that proportional error is almost absent (0.9651, very close to 1) and the constant error (278 bp) is lower than the value that corresponds to 1 standard deviation (SD) of our method (see next section).

The observed TAT systemic error could also be due to the fact that our method generates median telomere lengths values, while output of the TRF analysis consists of mode lengths values. This difference may, at least partially, account for the lack of even higher accuracy levels. Since the distribution of telomere lengths present in a cellular sample does not follow a normal distribution [25], the median telomere length (MTL) provides a more faithful representation of the sample.

### Precision Assessment

To determine the precision of TAT, 40 replicates of the same sample were processed and analyzed independently in 8 independent assays performed on different days by different testing personnel. The replicates were randomly seeded and analyzed in different positions in the plates to verify that any possible ‘position effect’ on the plate is accounted for and did not affect the results. The TAT standard deviation was defined as the square root of the total variance. The total variance was defined by the formula

$$ {\sigma}_{total}^2={\sigma}_{intra- run}^2+{\sigma}_{intra day}^2+{\sigma}_{interday}^2 $$

where the intra-run variance was the average variance of the 8 plates analyzed, the intraday variance was the average of the variances of the 4 days when analyses were performed, and the inter-day variance was the variance associated to the 4 day average results (see Materials and Methods for definitions). The results of our independent assays were that the intra-run variance was 162,021, the intraday variance was 7106 and the inter-day variance was 37,396. The TAT standard deviation was defined as the square root of the total variance, resulting in a SD of 454 base pairs.

### Tat LOD

In TAT the fluorescent signal emitted from the fluorophores used to label the object of interest in our specimen is quantified. For this reason, the LOD of TAT is intrinsically related to the detection limits of the image analysis system, and to how a fluorescence spot due to a specific telomeric hybridization signal is considered and analyzed. The image analysis system contained a dedicated script developed to perform the analysis of telomere staining on the different images (Acapella Software; Perkin Elmer) within the Opera® High-Content Screening System. Life Length uses the library of pre-tested scripts for the Acapella High Content Imaging and Analysis Software with modifications to meet the needs of TAT (See Materials and Methods for script settings).

A spot is identified as a defined region in the image that has higher intensity than its surrounding area. Spots can be searched for in a specified cellular region which in our case corresponds to the nuclear area, identified by DAPI staining. A scheme of the process and examples of the image analysis system are shown in Fig. 3a. The Opera Automatic Confocal Microscope detects and distinguishes up to 4095 different levels of fluorescence intensities (range 1–4095, expressed as gray levels). The baseline level of fluorescence intensities detected by the microscope even in the absence of fluorescent specimen is equal to 100 Gy levels. This is considered as background and therefore represents the LOD associated to the microscope sensitivity. However, these values vary slightly according to the parameters of the internal calibration curve run on each plate (standard curve cell lines are seeded in 8 replicates per cell line at different positions in the 384 well plate). Therefore, considering that our method SD is 454 bp, we could establish the LOD of TAT as ~ 800 bp corresponding to the calculated LOD (278 bp) + 1 SD (454 bp).

### Reportable Range and Reference Range

**Fig. 4:**
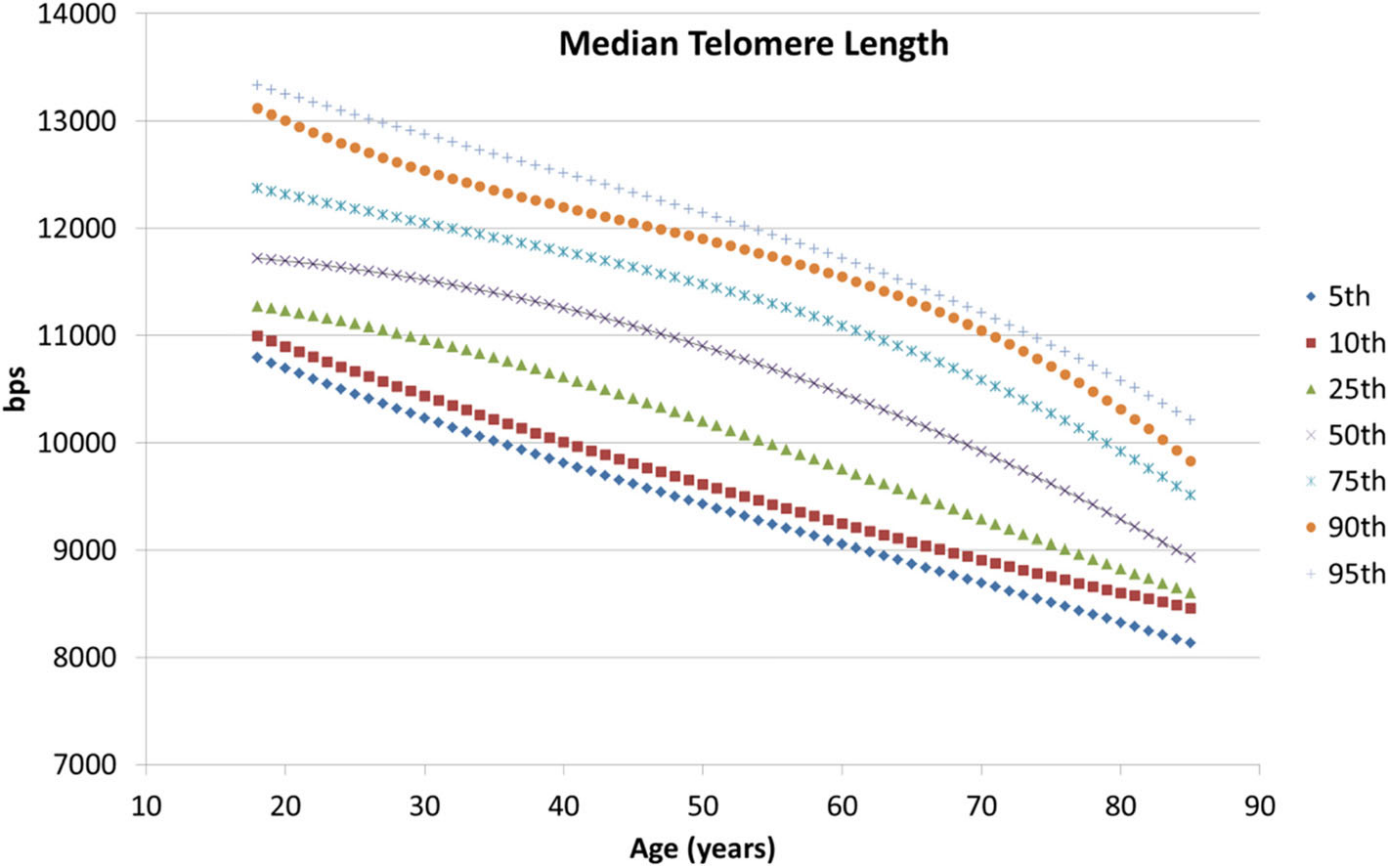
Median TL reference range. Median TL was measured in 406 healthy subjects ranging in age from 18 to 85 years. The 5th, 10th, 25th, 50th 75th and 95th percentiles are shown

Average human telomere length ranges from approximately 10 to 12 Kbp in newborns to 5 to 6 Kbp in subjects older than 60 years [26]. As demonstrated with the correlation with TRF, the reportable range of median telomere length values by TAT would be included in the range that spans the interval between our shortest telomere cell line (CO-154, 4.7 Kbp) and the longest one (REH, 14.4 Kbp) included in the assay. Since this range encompasses the telomere length reported for humans TAT can be applied to measurement of human telomeres in lymphocyte samples. To establish a reference range for telomere measurements by TAT in the human population, we tested blood from a sample cohort of 406 healthy subjects with an age range between 18 and 85 years. The results of median TL are shown in Fig. 4, where the different percentiles (5th, 10th, 25th, 50th 75th and 95th) are represented. These curves show the distribution of the median telomere length for each age and therefore, test samples results could be contrasted with these references to assess a given individual’s percentile range. Similar curves can be constructed modeling other relevant telomeric variables.

### Telomere Associated Variables

**Fig. 5:**
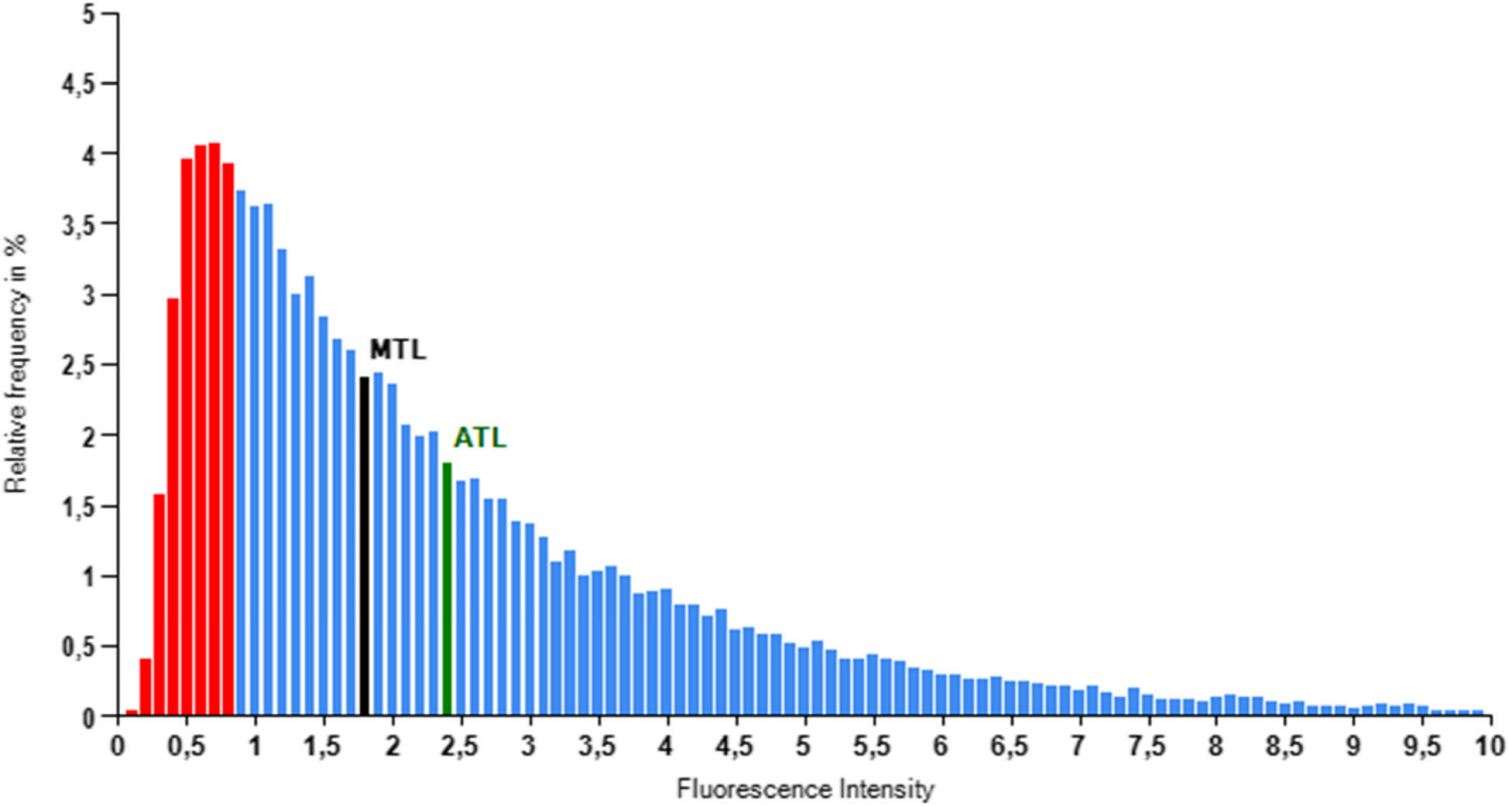
Example of telomeric histogram representative of telomere length distribution. This histogram is obtained for each sample analyzed by TAT. The red bar area indicates the 20th percentile. ATL, average telomere length and MTL, median telomere length

A telomeric histogram representative of telomere length distribution is obtained per each sample analysed by TAT, as shown in Fig. 5. Since the distribution of telomere length in the cells is asymmetrical, median telomere length (MTL) is a better representative value than average telomere length (ATL). The distribution of telomere lengths provided by TAT allows for a detailed statistical description of each sample in terms of descriptive statistics, TL percentiles, the percentage of telomeres comprised between two specific TL values in 500 bp intervals (ShortTel), the percentage of cells comprised between two specific TL values in 500 bp intervals (ShortCell), or dispersion statistics. The complete set of 223 telomere-associated variables (TAVs) is shown in Table 1. The variables for Percentile include 98 variables, ShortTel 80 variables, and ShortCell 40 variables, as indicated in the description of each of these variables in the table.

**Table 1:** Telomere-associated variables

Variable type	Variable name	Description
Descriptive statistics	Total Nuclei	Total number of nuclei in the sample.
	StDevPromedioI2	Standard deviation of the I2 median of the wells in a sample
	CoVPromedioI2	Variation Coefficient of the I2 median of the wells in a sample
	LengthI2Normalized	TL corresponding to the normalization of the median of intensities
	Length20Percentil	TL corresponding to the normalization of the 20th percentile of the intensities of a sample
	TamPromedio	Average nucleus size per sample
	MedianI2	Median of intensities per sample
	MedianI2Normalized	Normalization of the median of intensities per sample
	LengthAVG_I2	Average telomeric length of the telomeres in a sample
	SDMedianLength	Standard deviation of the median telomere lengths of the wells of a sample.
	CVMedianLength	Variation coefficient of the median TL of the wells of a sample
Telomere Length Percentiles	Percentil N	TL corresponding to the Nth percentile of a sample. Percentiles 1 and 100 are not included (up to 98 values)
Percentages of telomeric length values (ShortTel)	ShortTel N	Percentage of telomeres of a sample with a TL under the threshold N (80 values; i.e.: < 3 Kbp)
Percentages of cells with specific telomere values (ShortCell)	ShortCell N	Percentage of cells of a sample whose telomeres average a length under the threshold N (40 values)
Dispersion	MAD-I2	Median Absolute Deviation of the intensities of a sample
	P1–99-Kpbs	Difference between the 99th percentile of the TLs of a sample and the 1st percentile of the telomere lengths of that sample.
	P75–25-Kpbs	Interquartile of the TLs of a sample.

### Pilot Study: TAT for Large Population Studies

A pilot study was designed to analyze the feasibility of TAT for the analysis of large populations. The goal was to establish, using TAT, general age and gender-dependent trends of TL, and percentage of short telomeres. These baseline values in healthy individuals will be used as reference for future population-level studies. This pilot study was associated to the EU-funded project Oncocheck (Horizon2020 SME Instrument 738,707; www.oncocheck.eu), which had as the main objective the assessment of the clinical value of TAVs as cancer biomarkers. Oncocheck included cohorts of cancer patients and controls. Blood samples were obtained during regular, pre-scheduled blood tests or programmed interventions at the hospital. The demographic and clinical data associated with each patient were gathered from clinical records and recoded in ad hoc designed electronic notebooks. Control data was obtained from self-reported questionnaires.

In this pilot study a total of 8135 samples derived from the healthy patients (controls) were included and processed with TAT. These samples were obtained with the participation of 32 hospitals in Spain and from internal R&D studies over a period of 3 years. Each participant signed the pertinent informed consent for the use of the biological sample in the study. All samples met the requirements of having associated complete clinical records, quality control, and adequate reportable range. Human blood (10 ml) was collected in barcoded K2 EDTA tubes and keep between 2 and 8 °C for a maximum of 48 h before PBMC extraction by the Ficoll-Histopaque method (Fig. 1).

Of the 8135 samples, 4809 (59.1%) derived from male patients (mean [±SD] age of 49.2 ± 18.8 years) and 3326 (40.9%) from female patients (mean [±SD] age of 46.9 ± 18.9 years). A minimum of 1000 nuclei were analyzed from each sample plated in quintuplicate wells. Median telomere length was calculated for each of the replicates (5 for each sample). Only those samples with a variation coefficient (CV) < 10% of their replicates were analyzed as predefined for quality control purposes.

**Fig. 6:**
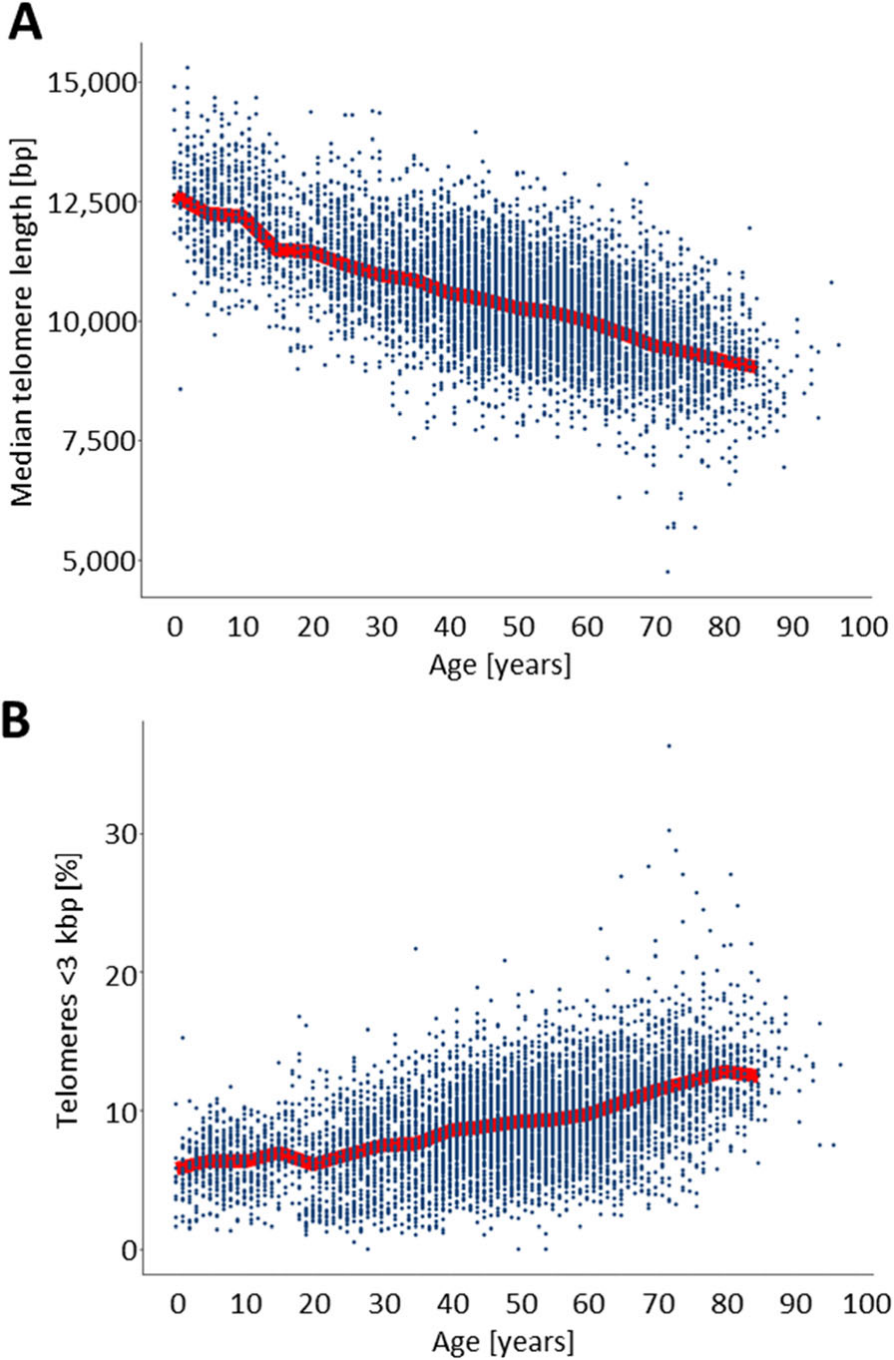
Distribution of telomere-associated variables as a function of patient age. **a**. Median telomere length; **b**. Telomeres < 3 kbp. For each graph the full set of patients (*n* = 8135, ages 0 to 97 years) were represented by dots and the trend of the median values for each variable superimposed (red line). The trendline (calculated in 5-year intervals) included only patients up to 85 years

**Fig. 7:**
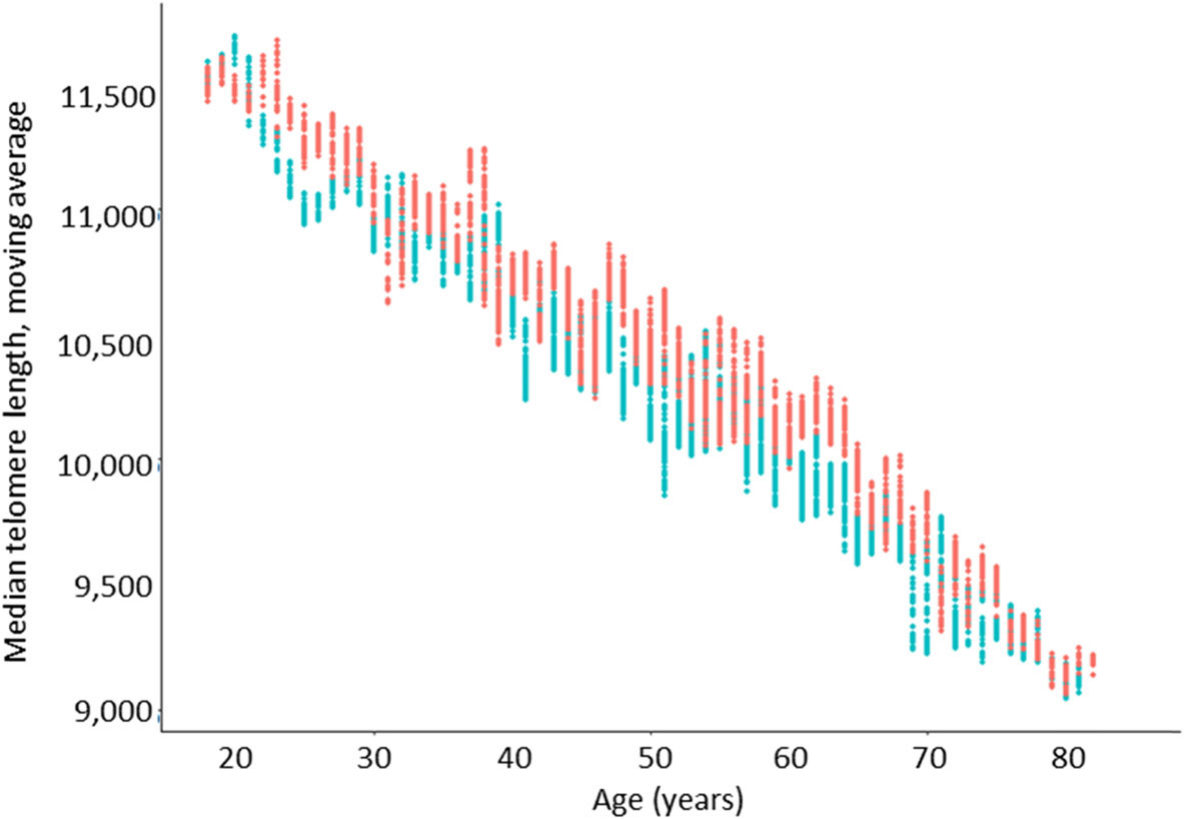
Distribution of median telomere length (simple forward moving average) by patient gender (Female, red; men, blue) and age. Data plotted was derived from the dataset of patients between 18 and 85 years old with outliers removed (*n* = 7325, men = 4310, women = 3015)

When considering the total population in our database (*n* = 8135; ages 0–97), we observed that the median TL decreased with age from ~ 13,000 bp to ~ 9000 bp (Fig. 6a). The percentage of short telomeres < 3 Kbp also increased with age (Fig. 6b). Telomere attrition by gender and age showed that women have, on average, larger telomeres than men of the same age (Fig. 7). The median TL attrition rate was constant for all ages (Fig. 7 and Table 2). However, for the age range from 48 to 57 years, males appeared to have a slower telomeric attrition rate (83 bp versus 241 bp in females).

**Table 2:** Telomere attrition by gender and age range

Age range	Gender	n^1^	Telomere attrition (bp)^2^ (mean ± SD)	*p*
18–85	M & F	7325	173 ± 3	< 0.001
18–37	M	600	222 ± 9	0.015
	F	659	148 ± 17	
38–47	M	899	149 ± 62	0.010
	F	653	160 ± 2	
48–57	M	1178	83 ± 28	< 0.001
	F	727	241 ± 49	
58–67	M	942	256 ± 4	< 0.001
	F	531	217 ± 23	
68–85	M	691	134 ± 43	0.016
	F	445	144 ± 26	

^1^ Outliers not included

^2^ Telomere length calculated over 5-year periods

## Discussion

Here we present the analytical validation data supporting a high-complexity, laboratory-developed test to be use with human samples, termed Telomere Analysis Technology (TAT), for the accurate determination of TL and other variables in circulating leukocytes. The principle of TAT is quantitative fluorescence in situ hybridization (Q-FISH), but it includes the use of a high-content screening microscope and a bioinformatics workflow. In Q-FISH, the complementarity of the fluorescent-labelled probe to the analyzed DNA sequence (telomeric repeats) renders the signal intensity quantifiable which is proportional to the length of the sequence [16]. Individual telomere intensities are compiled and analyzed to obtain median values of the different telomere lengths in the sample.

In our validation of TAT a correlation of 0.99 was established between TAT fluorescence intensity and telomere lengths values calculated by TRF, considered the reference “standard method” for telomere length distribution analysis [11, 27]. This correlation was established using 6 different lymphocyte cell lines with similar biological properties to PBMCs, a cell type routinely analyzed in many cancer studies [28, 29]. Serial analysis of median TL values of a human lymphocyte sample in different runs, days and plate positions define a TAT random error parameters (Standard Deviation, Variance) and indicated that TAT has a standard deviation of 454 base pairs. We established a LOD of 800 bp and demonstrated high specificity of the probe for the telomere repeats as seen in the image acquisition (Fig .3a) and reported elsewhere (Neumann et al. 2013 and Gazzaniga et al., 2015). Analysis of median TL of 6 cell lines that cover the human median reportable range defined its lower limit at 4700 bp and upper limits at 14,400 bp. Additional, reference cell lines with longer median TL could in the future be used to extend this range for animal/veterinary studies.

In our validation analysis we have used PBMCs as the reference cell type on which to test telomere measuring techniques. However, TAT could be easily adapted to the analysis of telomeres in any single-cell suspension including disaggregated OCT-frozen and paraffined embedded tumor biopsies. In this regard, TAT could be considered a platform for the general analysis of telomeres in any type of cells that can be assessed individually making it broadly applicable to in vitro and clinical studies. For example, a recent study demonstrated the use of TAT for the analysis of TL and its possible relationship with cell senescence in cultured chondrocytes [30].

In a pilot study using TAT we have looked at specific TAVs to examine their relationship with age and sex in a population-based cohort of children and adults. Our results are consistent with previous studies showing telomere attrition with age and a gender difference in this trend [24, 31]. Although the key factors for the sex difference are not fully understood, it has been suggested that the rates of attrition are largely similar for the two sexes, but that the sex difference in TL is established during childhood (age 1–20) [32]. Other possibilities, such a regulation of telomerase activity by estrogen, have also been explored [33]. Clearly more studies are needed to establish if there are gender differences in TL dynamics, as these effects could be involved in observed differences in resistance to ageing-related atherosclerotic cardiovascular disease and longevity. The use of an analytically validated technology to perform large population studies is critical to draw conclusions on complex diseases in future studies. In biomarker development this needs to be done beforehand in order to ensure standardization in the quality of the data. In addition, the ability to obtain over 200 telomere-related variables allows for the development of machine learning exercises.

Although telomere attrition occurs with increased age in humans, cross-sectional association studies with environmental or social variables such as smoking, the consumption of certain drugs, obesity, or stress should be evaluated with caution until longitudinal, prospective, studies can be performed [9, 20, 28, 34]. Many studies have disregarded the fact that, to mitigate measurement error, very large number of individuals must be evaluated to detect the influence of specific factors. The data generated in this pilot study is valuable to define parameters and ranges of the healthy population and is consistent with previous studies. This population-level study will be valuable in future comparative studies with populations at risk.

In the last two decades telomeres have emerged as biomarkers to assess several conditions and the cumulative influence of environmental and behavioral factors on complex disease risk. However, due to the large number variables affecting TL, the number of confounding factors that need to be evaluated in population studies also increases exponentially. For this reason, it becomes imperative the development of robust measurement techniques for TL that can look at very large numbers of cells and in many individuals in each cohort, and with a low intrinsic variability rate. In this regard, the technology described here, TAT, allows for the examination of many telomere associated variables (TAVs) reliably and reproducibly, and can be run in a high throughput format. This is in contrast with other telomere measurement methods such as qPCR, where only relative average telomere length can be determined; STELA, a gel-based technology of limited throughput; or Flow-FISH, which provides values of average TL in cells and not individual telomere length determinations [10].

TAVs provide telomere length determination with the granularity that other methods lack. As such, it is important to underline that samples with identical average telomere lengths as determined by qPCR can have very different percentages of short telomeres or a lower or higher proportion of cells with long telomeres; or yet, a narrow or wider dispersion values. This is critical to define the population characteristics of the subject, that can then be compared with patients at risk of having age-related diseases such as cancer, and to define if these variables or their combination influence diagnosis or prognosis.

## Conclusion

In summary, the innovations of TAT with respect to HT Q-FISH and other technologies for the analysis of telomeres are:

- The analytical validation and subsequent CLIA-88 and ISO15189 accreditation as a laboratory-developed test, which allows for the use of TAT in clinical settings (not for research only).
- Newly-developed analytical software workflow within TAT permits robust, absolute measurements (in base pairs) of TL.
- The robustness of TAT allows for statistical analysis and the determination of many telomere-associated variables (TAVs) not included until now in any scaled-up telomere measurement technique.
- TAT integrates methodological improvements such as the use of liquid handling robotics for cell seeding and addition/removal of reagents, or the use of HT imaging.

We anticipate that this powerful tool will help to expand our knowledge on the role of TL in human disease in the context of human population and epidemiological studies and will pave the way to develop diagnostic and prognostic biomarkers for telomere damage associated to age-related diseases.

## Methods

To evaluate the performance of TAT, analytical validation studies were performed in the laboratories of Life Length SL (Madrid, Spain) within the scope of CLIA (99D2112462) and ISO 15189 quality standards. Life Length, certified by the Spanish Ministry of Health as a Clinical Diagnostic Laboratory, defined the general assay systems and SOP. The analytical validation plan consisted of a set of studies aimed to establish the analytical performance of TAT, including determining its accuracy, precision, LOD, specificity, reportable range, and reference range. Additionally, a pilot study was carried out to assess the performance and value of TAT with clinical samples and in large population studies.

### Cell Lines

To evaluate analytical performance of TAT, we used commercially available lymphocyte cell lines (Table 3). Lymphocytes are generally in interphase (G0 phase of the cell cycle) and therefore the TL values obtained corresponded to cells with a G0 DNA content. TAT was developed initially for the analysis of PBMCs, which are lymphocytes derived from whole blood isolated by a density gradient that separates lymphocytes form granulocytes and erythrocytes. The cell lines used were cultured in Life Length’s laboratories using vendor-recommended conditions (RPMI 1640 growth media) supplemented with 10% FBS (v/v), 2 mM glutamine, and Penicillin/Streptomycin 0.1 mg/ml. The REH cell line medium was also supplemented with 1 mM NaPyr and 10 mM HEPES. Cultured cells were harvested and frozen in 90% FBS (v/v) supplemented with 10% (v/v) DMSO.

**Table 3:** Cells cultures used in this study

Cell name	Species	Tissue of Origin	Disease Status	Cell Type	Biosafety level	Source	Reference	Lot Number
CEM	Human	Peripheral blood	Acute lymphoblastic leukemia	T lymphoblast	1	ATCC	ATCC-CCL-119	59,057,235
C0106	Human	Blood	Normal	Lymphoblast	2	ECACC	ECACC-91071212	HB1489
C0126	Human	Blood	Normal	Lymphoblastoid	2	ECACC	ECACC-93020411	HB6380
C0154	Human	Blood	Normal	Lymphoblast	2	ECACC	ECACC-93012805	HB6343
RAJI	Human	Blood	Burkitt’s lymphoma	B lymphocyte	2	ATCC	ATCC-CCL-86	60,131,961/ 63,905,419
REH	Human	Blood	Acute lymphocytic leukemia (nonT; nonB)	Lymphoblast	1	ATCC	ATCC-CRL-8286	59,429,141

Abbreviations: *ATCC* Americal type culture collection; *ECACC* European Collection of Authenticated Cell Cultures

### TAT Workflow and HT Q-FISH Procedure

Cell lines frozen in liquid nitrogen were thawed (in RPMI1640 + 1% FBS [v/v]) and cell counts and cellular viability determined with an EVE™ Automated Cell Counter. Aliquots with viability lower than 60% were discarded. Cells were seeded in clear bottom, black-walled, 384-well plates at the density of 15,000 cells per well with 5 replicates of each sample and 8 replicates of each control cell line. After allowing attachment cells were fixed with methanol/acetic acid (3/1, vol/vol) and digestion with pepsin, the cells were washed several times in order to remove the components of seeding medium and serum that could cross-react with the probe in a non-specific way. Cell seeding, addition and removal of the reagents and washes are conducted using robots Freedom EVO2® and Fluent® by TECAN. Following the washings, the cells were hybridized in situ with a fluorescent Peptide Nucleic Acid (PNA) probe that recognizes three telomere repeats (Alexa488-OO-CCCTAACCCTAACCCTAA, Panagene Inc., South Korea). The PNA probe is a nucleotide analogue capable of binding to nucleic acids in a sequence-specific manner obeying the Watson-Crick base pairing rules. In PNA, the sugar phosphate backbone of nucleic acids has been replaced by a synthetic peptide backbone keeping the distances between bases exactly the same as in DNA or RNA; increasing affinity and stability. The hybridization step used a buffer that containing a blocking reagent that prevented non-specific binding (Roche Cat No. 11096176001), and extensive washes after the hybridization ensured removal of excess probe and weak, non-specific binding. After washing and 4′,6-diamidino-2-phenylindole (DAPI) staining, the wells were filled up with PBS and the plate stored at 4 °C until analyzed on the microscope.

In quantitative fluorescence microscopy interference with the detection and therefore interpretation of the fluorescence signal depends primarily on the specificity of the probe and the correct hybridization protocol. The PNA telomeric probe used in TAT is a commercially available molecule and its specificity has been extensively reported in quantitative FISH protocols for telomere hybridization [35–38]. Also, control cells were hybridized with a PNA telomeric anti-sense probe. This probe has the same nucleotide composition, yet it is not complementary to the telomeric repeat and therefore does not bind to the telomeres. The average number of spots identified in these negative controls is considered as background and therefore subtracted from the analysis of samples.

### High-Content Screening (HCS) and Image Acquisition

Quantitative image acquisition and analysis was performed on a High Content Screening Opera System (Perkin Elmer Inc., USA), using the Acapella software, Version 1.8 (Perkin Elmer, Inc., USA). The software is composed of different scripts comprising algorithms whose function is identifying different image elements (i.e. real fluorescent spots) and analyzing their features (i.e. intensity). The Acapella Scripts allows high quality images acquired with the Opera® High Content Screening System to turn image data quickly and easily into unbiased, statistically relevant results.

Images were captured using a 40 × 0.95 NA water immersion objective. UV (358 nm) and 488 nm excitation wavelengths were used to detect the DAPI and A488 signals respectively. With constant exposure settings, 15 independent images were captured at different positions for each well. Next, the nuclei images were used to define the region of interest for each cell, measuring telomere fluorescence intensity of the A488 image in all of them. The results of intensity for each foci were exported onto the microscope software (Columbus 2.4; Perkin Elmer, Inc., USA). Relative fluorescence intensity was defined as the ratio between the median intensity value of the well (I2) and the average of the median intensities of the control wells in the same plate. This normalization allowed minimization of inter-plate variability and therefore was used to compare results obtained in different runs. The intensities of fluorescence were translated to base pairs through a standard regression curve which was generated using control cell lines with known telomere length.

The Nuclei Detection Script ensured that only spots located inside the nucleus were considered for analysis, which meant that a spot was detected only if its area was also positive for DAPI staining. This eliminated any possible interference of extra-nuclear (i.e., cytoplasmic) signals. The script consisted of the following parameters: Threshold Adjustment (threshold tuning for the initial thresholding of nuclei, with a range 0–3.0, set to 1.5); Minimum Nuclei Distance (minimum allowed distance between nuclei centers, set to 7 pixels); Nuclear Splitting Adjustment (controls splitting of close nuclei, set to 7 pixels); Individual Threshold Adjustment (size of nuclei, with a range from 0 to 1.0, set to 0.15); Minimum Nuclear Area (minimum allowed area for nuclei, set to 70 pixels); and Minimum Nuclear Contrast (minimum allowed contrast for nuclei, removing objects with contrast lower than a limit, range from 0 to 1.0, set to 0.1).

The Spot Detection Script was set based on the following parameters: Spot Minimum Distance (minimum allowed distance between two spot centers, set to 3 pixels); Spot Peak Radius (radius of the disk where the spot peak was calculated, set to 0 pixels so that peak intensity corresponded to the intensity of the most intense pixel of the spot); Spot Reference Radius (radius of reference region around the intensity maximum, set to 3.1 pixels, it specified the maximum radius of the region around the peak where the spot could be defined); Spot Minimum Contrast (minimum allowed contrast between spot peak intensity and the reference intensity, set to 0.25); and Spot Minimum to Cell Intensity (minimum allowed intensity ratio between spot peak intensity and the average intensity of the object to which the spot belongs, set to 1).

### Bioinformatics and Data Analysis

Image data from the High Content Screening Opera System was analyzed with Acapella® software (Perkin Elmer). Data obtained from Acapella® was analyzed by the Plate Analyzer software of Life Length SL. Further data unification and formatting was performed with Pentaho (Hitachi Vantara).

The Knime platform (KNIME AG, Zurich, Switzerland) and R (3.4.2) were used for clinical data analysis and modeling, and ggplot2 (3.1.0) for data visualization.

### Terminal Restriction Fragment Analysis

TRL was used to compare quantitatively results derived from TAT. In brief, genomic DNA extracted from the cell lines was digested using a cocktail of frequent-cutting restriction enzymes that lack recognition sites in the telomeric and subtelomeric regions [39]. The intact telomeres (and some sub-telomeric DNA) from all chromosomes were then resolved by agarose gel electrophoresis, with the telomeric fragments being visualized by in-gel hybridization using the DIG-labelled telomere 18-mer probe (CCTAAA)_3_ (Sigma Aldrich). The varying lengths of telomeres were assessed by comparison to a DNA ladder comprised of known fragment sizes.

## Data Availability

Data presented in this manuscript is available upon request.

## Abbreviations

CLIA: Clinical laboratory improvement amendments
DAPI: 4′,6-diamidino-2-phenylindole
DMSO: Dimethyl sulfoxide
FBS: Fetal bovine serum
FISH: Fluorescent in situ hybridization
HCS: High-content screening
HEPES: 4-(2-hydroxyethyl)-1-piperazineethanesulfonic acid
HT: High throughput
IGHV: Immunoglobulin heavy chain variable
LOD: Limits of detection
MTL: Median telomere length
PBMC: Peripheral blood mononuclear cells
PCR: Polymerase chain reaction
PNA: Peptide nucleic acid
Q-FISH: Quantitative fluorescent in situ hybridization
qPCR: Quantitative polymerase chain reaction
RPMI: Roswell Park Memorial Institute
SD: Standard deviation
SOP: Standard operating procedure
TAT: Telomere Analysis Technology
TAV: Telomere-associated variable
TL: Telomere length
TRF: Terminal restriction fragment

## References

[1] Blackburn EH, Greider CW, Szostak JW (2006). Telomeres and telomerase: the path from maize, Tetrahymena and yeast to human cancer and aging. Nat Med.

[2] Blackburn EH (2005). Telomeres and telomerase: their mechanisms of action and the effects of altering their functions. FEBS Lett.

[3] Yui J, Chiu CP, Lansdorp PM (1998). Telomerase activity in candidate stem cells from fetal liver and adult bone marrow. Blood.

[4] Sahin E, Depinho RA (2010). Linking functional decline of telomeres, mitochondria and stem cells during ageing. Nature.

[5] Cesare AJ, Reddel RR (2010). Alternative lengthening of telomeres: models, mechanisms and implications. Nat Rev Genet.

[6] Zakian VA (2012). Telomeres: the beginnings and ends of eukaryotic chromosomes. Exp Cell Res.

[7] Frenck RW, Blackburn EH, Shannon KM (1998). The rate of telomere sequence loss in human leukocytes varies with age. Proc Natl Acad Sci U S A.

[8] Epel ES, Blackburn EH, Lin J, Dhabhar FS, Adler NE, Morrow JD (2004). Accelerated telomere shortening in response to life stress. Proc Natl Acad Sci U S A.

[9] Aviv Abraham, Shay Jerry W. (2018). Reflections on telomere dynamics and ageing-related diseases in humans. Philosophical Transactions of the Royal Society B: Biological Sciences.

[10] Lai Tsung-Po, Wright Woodring E., Shay Jerry W. (2018). Comparison of telomere length measurement methods. Philosophical Transactions of the Royal Society B: Biological Sciences.

[11] Kimura M, Stone RC, Hunt SC, Skurnick J, Lu X, Cao X (2010). Measurement of telomere length by the southern blot analysis of terminal restriction fragment lengths. Nat Protoc.

[12] Baird DM, Rowson J, Wynford-Thomas D, Kipling D (2003). Extensive allelic variation and ultrashort telomeres in senescent human cells. Nat Genet.

[13] Lin J, Smith DL, Esteves K, Drury S (2019). Telomere length measurement by qPCR - summary of critical factors and recommendations for assay design. Psychoneuroendocrinology.

[14] Aubert G, Hills M, Lansdorp PM (2012). Telomere length measurement-caveats and a critical assessment of the available technologies and tools. Mutat Res.

[15] Baerlocher GM, Vulto I, de Jong G, Lansdorp PM (2006). Flow cytometry and FISH to measure the average length of telomeres (flow FISH). Nat Protoc.

[16] Canela A, Vera E, Klatt P, Blasco MA (2007). High-throughput telomere length quantification by FISH and its application to human population studies. Proc Natl Acad Sci U S A.

[17] Ourliac-Garnier I, Londono-Vallejo A (2017). Telomere length analysis by quantitative fluorescent in situ hybridization (Q-FISH). Methods Mol Biol.

[18] Hemann MT, Strong MA, Hao LY, Greider CW (2001). The shortest telomere, not average telomere length, is critical for cell viability and chromosome stability. Cell..

[19] Rode L, Nordestgaard BG, Bojesen SE (2015). Peripheral blood leukocyte telomere length and mortality among 64,637 individuals from the general population. J Natl Cancer Inst.

[20] Wang Q, Zhan Y, Pedersen NL, Fang F, Hagg S (2018). Telomere length and all-cause mortality: a meta-analysis. Ageing Res Rev.

[21] Quintela-Fandino M, Soberon N, Lluch A, Manso L, Calvo I, Cortes J (2017). Critically short telomeres and toxicity of chemotherapy in early breast cancer. Oncotarget.

[22] Erdmann N, Liu Y, Harrington L (2004). Distinct dosage requirements for the maintenance of long and short telomeres in mTert heterozygous mice. Proc Natl Acad Sci U S A.

[23] Blasco MA, Lee HW, Hande MP, Samper E, Lansdorp PM, DePinho RA (1997). Telomere shortening and tumor formation by mouse cells lacking telomerase RNA. Cell..

[24] Harley CB, Futcher AB, Greider CW (1990). Telomeres shorten during ageing of human fibroblasts. Nature.

[25] Lansdorp PM, Verwoerd NP, van de Rijke FM, Dragowska V, Little MT, Dirks RW (1996). Heterogeneity in telomere length of human chromosomes. Hum Mol Genet.

[26] Calado RT, Dumitriu B (2013). Telomere dynamics in mice and humans. Semin Hematol.

[27] Mender I, Shay JW. Telomere Restriction Fragment (TRF) Analysis. Bio Protoc. 2015;5(22):e1658. 10.21769/bioprotoc.1658 PMC4972328 27500189

[28] Zhang X, Zhao Q, Zhu W, Liu T, Xie SH, Zhong LX (2017). The Association of Telomere Length in peripheral blood cells with Cancer risk: a systematic review and meta-analysis of prospective studies. Cancer Epidemiol Biomark Prev.

[29] Mons U, Muezzinler A, Schottker B, Dieffenbach AK, Butterbach K, Schick M (2017). Leukocyte telomere length and all-cause, cardiovascular disease, and Cancer mortality: results from individual-participant-data meta-analysis of 2 large prospective cohort studies. Am J Epidemiol.

[30] Lopez-Alcorocho JM, Guillen-Vicente I, Rodriguez-Inigo E, Guillen-Vicente M, Fernandez-Jaen TF, Caballero R (2019). Study of telomere length in Preimplanted cultured chondrocytes. Cartilage.

[31] Gardner M, Bann D, Wiley L, Cooper R, Hardy R, Nitsch D (2014). Gender and telomere length: systematic review and meta-analysis. Exp Gerontol.

[32] Dalgard C, Benetos A, Verhulst S, Labat C, Kark JD, Christensen K (2015). Leukocyte telomere length dynamics in women and men: menopause vs age effects. Int J Epidemiol.

[33] Bayne S, Jones ME, Li H, Liu JP (2007). Potential roles for estrogen regulation of telomerase activity in aging. Ann N Y Acad Sci.

[34] Casagrande S, Hau M (2019). Telomere attrition: metabolic regulation and signalling function?. Biol Lett.

[35] Meena JK, Cerutti A, Beichler C, Morita Y, Bruhn C, Kumar M (2015). Telomerase abrogates aneuploidy-induced telomere replication stress, senescence and cell depletion. EMBO J.

[36] M'Kacher R, Maalouf EE, Ricoul M, Heidingsfelder L, Laplagne E, Cuceu C (2014). New tool for biological dosimetry: reevaluation and automation of the gold standard method following telomere and centromere staining. Mutat Res.

[37] Gazzaniga FS, Blackburn EH (2014). An antiapoptotic role for telomerase RNA in human immune cells independent of telomere integrity or telomerase enzymatic activity. Blood.

[38] Neumann AA, Watson CM, Noble JR, Pickett HA, Tam PP, Reddel RR (2013). Alternative lengthening of telomeres in normal mammalian somatic cells. Genes Dev.

[39] Jenkins FJ, Kerr CM, Fouquerel E, Bovbjerg DH, Opresko PL. Modified Terminal Restriction Fragment Analysis for Quantifying Telomere Length Using In-gel Hybridization. J Vis Exp. 2017;(125):56001. 10.3791/56001 PMC5612054 28715381

